# Cytogenetic and molecular analyses of 291 gastrointestinal stromal tumors: site-specific cytogenetic evolution as evidence of pathogenetic heterogeneity

**DOI:** 10.18632/oncotarget.28209

**Published:** 2022-03-07

**Authors:** Ludmila Gorunova, Kjetil Boye, Ioannis Panagopoulos, Jeanne-Marie Berner, Bodil Bjerkehagen, Ivar Hompland, Ingvild Lobmaier, Toto Hølmebakk, Tarjei S. Hveem, Sverre Heim, Francesca Micci

**Affiliations:** ^1^Section for Cancer Cytogenetics, Institute for Cancer Genetics and Informatics, The Norwegian Radium Hospital, Oslo University Hospital, Oslo, Norway; ^2^Department of Oncology, The Norwegian Radium Hospital, Oslo University Hospital, Oslo, Norway; ^3^Department of Tumor Biology, The Norwegian Radium Hospital, Oslo University Hospital, Oslo, Norway; ^4^Department of Pathology, The Norwegian Radium Hospital, Oslo University Hospital, Oslo, Norway; ^5^Institute of Clinical Medicine, Faculty of Medicine, University of Oslo, Oslo, Norway; ^6^Institute of Oral Biology, Faculty of Dentistry, University of Oslo, Oslo, Norway; ^7^Section for Applied Informatics, Institute for Cancer Genetics and Informatics, The Norwegian Radium Hospital, Oslo University Hospital, Oslo, Norway

**Keywords:** gastrointestinal stromal tumor, chromosome aberrations, intratumor heterogeneity, *KIT* mutations, *PDGFRA* mutations

## Abstract

Gastrointestinal stromal tumor (GIST) is a mesenchymal neoplasm with variable behavior. An increased understanding of the tumor pathogenesis may improve clinical decision-making. Our aim was to obtain more data about the overall chromosome aberrations and intratumor cytogenetic heterogeneity in GIST. We analyzed 306 GIST samples from 291 patients using G-banding, direct sequencing, and statistics. Clonal chromosome aberrations were found in 81% of samples, with 34% of 226 primary tumors demonstrating extensive cytogenetic heterogeneity. 135 tumors had simple (≤5 changes) and 91 had complex (>5 changes) karyotypes. The karyotypically complex tumors more often were non-gastric (*P* < 0.001), larger (*P* < 0.001), more mitotically active (*P* = 0.009) and had a higher risk of rupture (*P* < 0.001) and recurrence (*P* < 0.001). Significant differences between gastric and non-gastric tumors were found also in the frequency of main chromosome losses: of 14q (79% vs. 63%), 22q (38% vs. 67%), 1p (23% vs. 88%), and 15q (18% vs. 77%). Gastric *PDGFRA*-mutated tumors, compared with gastric *KIT*-mutated, had a lower incidence of 22q losses (18% vs. 43%) but a higher rate of 1p losses (42% vs. 22%). The present, largest by far karyotypic study of GISTs provides further evidence for the existence of variable pathogenetic pathways operating in these tumors’ development.

## INTRODUCTION

Gastrointestinal stromal tumor (GIST), the most common primary mesenchymal neoplasm of the gastrointestinal tract, accounts for 2–3% of all gastric malignancies [1, 2]. GISTs are characterized by variable behavior and differentiation towards the interstitial cells of Cajal [1, 2]. The tumors are generally immunopositive for CD117 (KIT) and DOG1 [3, 4].

Approximately 60–70% of GISTs occur in the stomach, 20–30% in the small intestine, 5% in the colon and rectum, and 1% in the esophagus [2]. On rare occasions, they form solitary masses (extragastrointestinal tumors) in the omentum or mesentery [2]. GISTs are mainly sporadic and occur in older adults (median age, 60–65 years) without gender predilection [2]. Complete resection is the treatment of choice in localized disease [5].

KIT proto-oncogene, receptor tyrosine kinase (*KIT*) and platelet derived growth factor receptor alpha (*PDGFRA*) oncogenic mutations have been detected in 80% and 10% of GISTs, respectively. They appear to be early events in GIST development [6, 7].

Additional genomic/chromosomal alterations are required for tumor progression. Such aberrations have been studied by different techniques: banding analysis, fluorescence *in situ* hybridization (FISH), comparative genomic hybridization (CGH), array CGH, and high-throughput sequencing [8–14].

A cytogenetic approach was, compared with other methods, infrequently applied to the study of genomic changes of GISTs, with karyotypes of less than 60 tumors having been reported [8]. This notwithstanding, cytogenetics provides a genome-wide overview of chromosome alterations while at the same time being informative about intratumor heterogeneity and the direction in which any clonal evolution may be proceeding. Furthermore, certain aberrations, such as balanced translocations, inversions, and telomeric associations, may not be detectable in studies using other methods.

Over more than two decades, we have as part of our diagnostic practice collected a consecutive series of GISTs analyzed cytogenetically and genotyped molecularly. Here, we report the accumulated chromosome and mutation data focusing on intratumor heterogeneity as well as cytogenetic evolution.

## RESULTS

The findings of cytogenetic analyses of 306 GIST samples from 291 patients together with molecular data on 254 tumors are presented in Supplementary Table 1.

Clonal chromosome aberrations were found in 248 samples and 237 cases (81% each). 220 (89%) of the karyotypically abnormal samples were near-diploid, 15 (6%) were near-triploid, 7 (3%) had both near-diploid and near-triploid/tetraploid cells, whereas 6 (2%) were in the 4n–6n ploidy range.

The set of primary tumors consisted of 226 abnormal cases. Their clinical and histopathological data are summarized in Table 1. Of the 226 GISTs, 149 (66%) showed one clone only, whereas the remaining tumors demonstrated clonal evolution. Specifically, 69 tumors (30%) displayed from 2 to 5 and 8 tumors (4%) from 6 to 23 karyotypically related clones. Unrelated clones (those with entirely disparate chromosome changes in cells from the same tumor) were found in a small proportion of cases (9%, 21/226).

**Table 1:** Clinical and histopathological data on 226 primary GISTs

Clinical and histopathological data	Number of patients (%)
Age (years)^a^	66 (23–93)
Sex	
Female	105 (46)
Male	121 (54)
Tumor location	
Esophagus	3 (1)
Stomach	181 (80)
Small intestine	33 (15)
Rectum	7 (3)
Extragastrointestinal	2 (1)
Tumor size (cm)^a^	5.0 (1.5–28.0)
Mitoses per 50 HPF^a,b^	2 (0–178)
Tumor rupture	
Yes	22 (10)
No	196 (87)
Not determined	8 (4)
Modified NIH risk criteria^c^	
Very low	7 (3)
Low	93 (41)
Intermediate	44 (19)
High	58 (26)
Metastatic	19 (8)
Not able to classify	5 (2)
Mutational analysis	
*KIT* exon 9	8 (4)
*KIT* exon 11	133 (59)
*KIT* exon 13	4 (2)
*KIT* exon 17	5 (2)
*PDGFRA* exon 12	3 (1)
*PDGFRA* exon 14	2 (1)
*PDGFRA* exon 18	31 (14)
No mutation detected	11 (5)
Not done	29 (13)
Preoperative systemic treatment	
No	208 (92)
Yes	18 (8)

^a^Values are median (range). ^b^HPF, high-power field of the microscope. ^c^Risk classification was performed at the time of primary tumor surgery or diagnosis. Abbreviation: NIH: National Institutes of Health.

The number of chromosome aberrations per tumor/clone varied from 1 to over 50. 135 tumors (60%) displayed simple karyotypes (defined as ≤5 chromosomal changes) whereas 91 (40%) tumors had complex ones (>5 changes). A comparison of these two groups (Table 2) reveals that the karyotypically complex tumors were more often non-gastric (*P* < 0.001), larger (*P* < 0.001), more mitotically active (*P* = 0.009) and had a higher risk of tumor rupture (*P* < 0.001) and recurrence (*P* < 0.001). In contrast, no significant differences in mutation status between these two cytogenetic groups were found.

**Table 2:** Associations between karyotypic complexity and clinicopathological variables in 226 primary GISTs

Clinicopathological variables	Karyotype		*P* value
	Simple (≤5 changes)	Complex (>5 changes)	
Age^a^	67 (25–87)	66 (23–93)	0.60
Sex			0.42
Female	66	39	
Male	69	52	
Tumor location			<0.001
Esophagus	1	2	
Stomach	122	59	
Small intestine	11	22	
Rectum	1	6	
Extragastrointestinal	0	2	
Tumor size (cm)^a^	4.2 (1.5–24.0)	7.0 (2.0–28.0)	<0.001
Mitoses per 50 HPF^a,b^	2 (0–53)	3 (0–178)	0.009
Tumor rupture			<0.001
Yes	6	16	
No	128	68	
Not determined	1	7	
Modified NIH risk criteria^c^			<0.001
Very low	7	0	
Low	66	27	
Intermediate	34	10	
High	20	38	
Metastatic	5	14	
Not able to classify	3	2	
Mutational analysis			0.31^d^
*KIT* exon 9	3	5	
*KIT* exon 11	75	58	
*KIT* exon 13	3	1	
*KIT* exon 17	5	0	
*PDGFRA* exon 12	3	0	
*PDGFRA* exon 14	2	0	
*PDGFRA* exon 18	18	13	
No mutation detected	4	7	
Not done	22	7	

^a^Values are median (range). ^b^HPF, high-power field of the microscope. ^c^Risk classification was performed at the time of primary tumor surgery or diagnosis; NIH, National Institutes of Health. ^d^ *P* value is calculated based on three categories of mutations: *KIT*, *PDGFRA*, and no mutation.

To get more accurate and representative data on the cytogenetic profile and specific aberrations of GISTs, we excluded tumors with incomplete karyotypes (cases 19, 93, 201, and 207) from further analyses. We also excluded changes found in unrelated clones, the karyotypic relevance of which is uncertain. These near-diploid clones displayed simple changes, most frequently -Y and +7, or occasionally balanced aberrations. Tumors with only such aberrations (cases 9, 51, 59, 104, 132, and 226) were also left out. The remaining 216 cases comprised 173 gastric and 43 non-gastric GISTs.

The identified chromosome abnormalities were both numerical and structural. Among the latter, balanced translocations were uncommon. Recurrent structural aberrations were the dicentric chromosome dic(19;19)(q13;q13) (7 cases), the isochromosomes i(1)(q10), i(8)(q10), and i(17)(q10), and the ring chromosomes r(16)(p13q24) and r(19)(p13q13) (2–3 cases each). 25 GISTs demonstrated clonal telomeric associations with up to 16 different tas per tumor.

Losses of chromosomes, entire or partial, were more common than gains in the vast majority of the near-diploid tumors/clones whereas in polyploid clones, particularly in near-triploid ones, the proportion of gains increased and could be comparable to losses.

**Figure 1:**
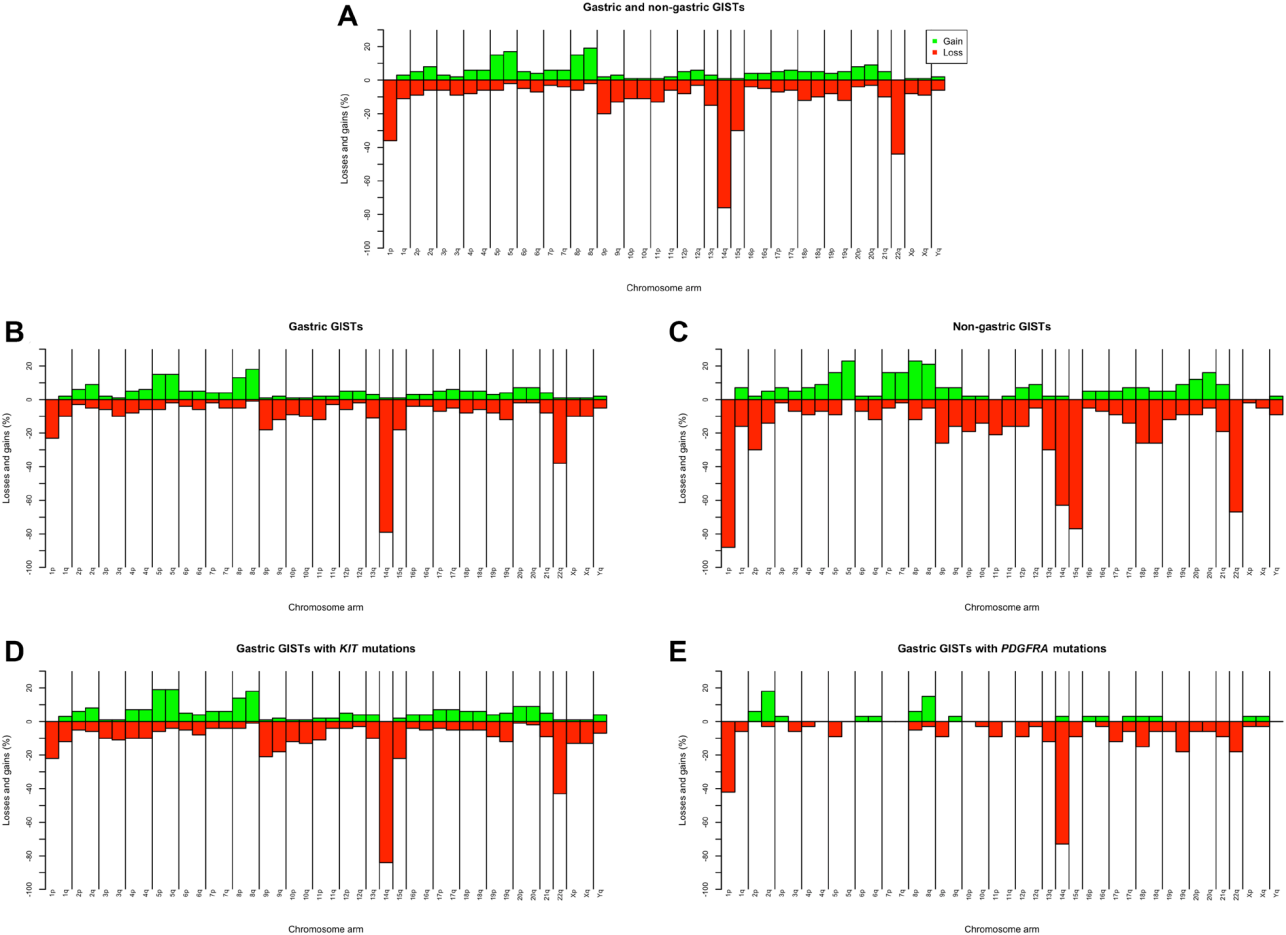
Chromosome imbalances identified by karyotyping in 216 primary both gastric and non-gastric GISTs (**A**); chromosome imbalances in 173 gastric (**B**) and 43 non-gastric (**C**) GISTs, and chromosome imbalances in 113 gastric GISTs with *KIT* mutations (**D**) and 33 gastric GISTs with *PDGFRA* mutations (**E**).

The frequencies of losses and gains in the 42 chromosome arms found in the 216 GISTs are summarized in Supplementary Table 2 and depicted in Figure 1A. In total, the most frequent losses affected chromosomes 14 (76%), 22 (44%), 1/1p (36%), and 15 (30%). In acrocentric chromosomes 14, 15, and 22, losses of the q-arm (p-arm changes were mostly untraceable) were partial only in less than 15% of cases, whereas whole chromosome losses predominated. In contrast, loss of an entire chromosome 1 was seen in less than 10% of tumors.

The most frequent loss, -14, was found in 29 GISTs as the sole abnormality. In addition, in 10 tumors displaying clonal evolution, a solitary -14 was present in the basic clone. The double loss -14 and -22 was the only change in 12 tumors, whereas in 6 cases such a clone was the basic one but was accompanied by karyotypic evolution.

Among whole chromosome gains, +5 (16%) and +8 (15%) were most common, followed by +2 and +20 (7% each) and +4 and +7 (6% each).

Four gastric (cases 31, 146, 178, and 282) and 2 esophageal (cases 57 and 288) GISTs demonstrated different karyotypic patterns; they were hyperdiploid (50–56 chromosomes) and displayed several (3 to 10) trisomies/tetrasomies alongside structural changes.

We further compared the cytogenetic profiles of gastric and non-gastric GISTs. The associations between tumor location and other clinicopathological variables are presented in Supplementary Table 3.

The patterns of imbalances in 42 chromosome arms of 173 gastric and 43 non-gastric GISTs are shown in Supplementary Table 4 and Figure 1B and 1C. Table 3 summarizes data on the associations between the four most frequent losses – of 1p, 14q, 15q, and 22q - on the one hand and tumor site on the other. Significant differences were found in the incidence of all these losses, with -14q being the most common in gastric and 1p- in non-gastric GISTs. Additionally, the non-gastric tumors showed statistically higher rates of the less frequent losses 2p- (30% vs. 3%; *P* < 0.001) and -13q (30% vs. 11%; *P* = 0.003).

**Table 3:** Associations between main chromosome losses, tumor location, karyotypic complexity, and genotype in GISTs

Loss of chromosome arm^a^	1p			14q			15q			22q		
	*n*	%	*P* value	*n*	%	*P* value	*n*	%	*P* value	*n*	%	*P* value
Location Gastric (*n* = 173) Non-gastric (*n* = 43)	40 38	23.1 88.4	<0.001	137 27	79.2 62.8	0.029	31 33	17.9 76.7	<0.001	65 29	37.6 67.4	<0.001
Karyotype (≤5 changes) Gastric (*n* = 137) Non-gastric (*n* = 16)	16 14	11.9 87.5	<0.001	112 8	81.8 50.0	0.007	9 12	6.6 75.0	<0.001	42 8	30.9 50.0	0.16
Genotype of gastric *KIT* (*n* = 113) *PDGFRA* (*n* = 33)	25 14	22.1 42.4	0.026	95 24	84.1 72.7	0.20	25 3	22.1 9.1	0.13	48 6	42.5 18.2	0.013

^a^Partial or complete loss.

To find out when in karyotypic evolution the said differences between gastric and non-gastric GISTs become evident, we selected for study tumors/clones with no more than 5 changes (i.e., with simple karyotypes). Altogether 55 chromosome imbalances were assessed in a total of 137 gastric and 16 non-gastric such tumors. As shown in Table 3, significant differences in the rate of losses of 1p, 14q, and 15q (but not 22q) were found between karyotypically simple gastric and non-gastric tumors/clones. Besides, non-gastric tumors with ≤5 changes had significantly higher incidence of 2p losses than did gastric tumors (20% vs. 0%; *P* < 0.001). For practically all the remaining imbalances (not shown), the values were below 10%, in fact 0% for half of them. Thus, the main differences in the cytogenetic profile between gastric and non-gastric GISTs emerge early in karyotypic evolution.

We further assessed a possible impact of mutation status on the cytogenetic profile of GISTs by comparing chromosome imbalances in the two main genotypic groups, *KIT*- and *PDGFRA*-mutated tumors. Since no *PDGFRA* mutations were identified in non-gastric GISTs in this study (Supplementary Table 3), and to avoid any site-specific effect in *KIT*-mutated tumors, only gastric tumors were compared.

Chromosome imbalances found in 42 chromosome arms of gastric 113 *KIT*- and 33 *PDGFRA*-mutated tumors are presented in Supplementary Table 5 and Figure 1D and 1E. Overall, *PDGFRA*-mutated tumors exhibited considerably fewer aberrations: in 46% (39/84) of all imbalances (losses or gains) the values were 0%, in contrast to only 2% (2/84) in *KIT*-mutated tumors. Table 3 shows that the frequencies of 14q and 15q losses did not differ significantly between *KIT*- and *PDGFRA*-mutated GISTs. In *PDGFRA*-mutated tumors, on the other hand, the rate of 1p losses was higher and 22q losses lower.

Altogether, 16 abnormal metastatic samples from 13 tumors (5 gastric and 8 non-gastric) were available for analyses. The majority of metastatic samples (11/16, 69%) were, like the primary tumor set, near-diploid, 4 (25%) were near-triploid, while one sample had 2n-4n clones. Half of the metastatic samples displayed clonal evolution in the form of 2–5 related clones whereas the clones in two samples were unrelated. All but one sample had complex karyotypes; however, that one simple karyotype carried a solitary +7 that probably was not representative of the tumor parenchyma.

On six occasions (cases 1a-c; 8a,b; 21b,c; 28a,b; 43a,b; and 78a-d) it was possible to compare primary tumors with their metastases. In general, metastatic samples resembled their matching primaries. Apart from that, the relationship between the genetic alterations of primary tumors and metastatic samples reflected three main evolutionary scenarios: First, the primary tumor and metastasis sometimes had practically identical or very similar aberrations (cases 43a,b and 28a,b). Second, the metastases could display a markedly higher degree of karyotypic complexity and/or heterogeneity than did the primary tumor (cases 1a-c and 78a-d). Third, and in contrast to the preceding scenario, the metastases sometimes exhibited less karyotypic complexity and/or heterogeneity (cases 8a,b and 21a,b). In three cases (1, 2, and 78), two metastatic samples were available for comparison; the synchronous metastases (cases 1b,c and 2a,b) showed closer karyotypic similarity to each other than did the consecutive samples (case 78c,d).

## DISCUSSION

Published cytogenetic data on GIST include less than 60 tumors with chromosome aberrations [3, 8], in contrast to information on several large series (around 50–200 cases) of tumors investigated by CGH/array CGH or high-throughput sequencing. The present, largest by far, study adds nearly 240 abnormal tumor samples, both gastric and non-gastric, to the existing karyotypic database, coupled with mutation data for the majority of them. Besides obtaining more knowledge about the general pattern of chromosome aberrations in GISTs, we also aimed at investigating cytogenetic heterogeneity in this tumor type and a comparison of the cytogenetic pathways taken by gastric and non-gastric GISTs.

It is generally accepted that whereas oncogenic *KIT* and *PDGFRA* mutations are necessary for the neoplastic transformation of GIST, additional somatic genomic alterations are required for tumor progression. Banding cytogenetics is one of the techniques best suited to monitor the stepwise acquisition of chromosome aberrations, i.e., clonal evolution, characteristic of this process. Indeed, detailed analysis revealed extensive cytogenetic heterogeneity in the present series, much higher than reported previously [8]. Clonal evolution was found in nearly 34% of GISTs, generally in the form of 2–5 clones/subclones (up to 23).

The acquisition of new aberrations did not always proceed in a linear manner but rather led to a variety of changes upon which further selection might work. By way of example, case 116b showed gain of an extra 8q arm occurring in one subclone through formation of der(8;13)(q10;q10), whereas in another subclone the same imbalance was the result of a dic(8;15)(p11;p11). In case 181, the initial changes -14 and -22 were followed in one subclone by a der(1)t(1;2)(q44;q11) leading to gain of 2q, while another subclone instead had a der(1)t(1;8)(q44;q22) causing gain of 8q.

Clonal telomeric associations were found in 25, mostly gastric, GISTs. In spite of the fact that telomeric associations do not allegedly involve loss of chromosome material, they may be the precursors of dicentric chromosomes. The latter are known to be prone to further rearrangements because of additional risk of breakage during mitosis. The karyotypes of some tumors demonstrated a strong association between clonal telomeric fusions of particular chromosomes and clonal dicentrics or derivatives of the same chromosomes with variable breakpoints. Thus, coexistence of clones with tas(11;19)(p15;q13), dic(11;19)(p11;q13), and der(11;19)(q10;q10) was seen in case 266. A particular dicentric chromosome, dic(19;19)(q13;q13), was found recurrently; a dic(19;19), no breakpoints were given, was reported in GIST previously [15].

In agreement with the findings of CGH/array CGH studies [9–11], chromosome losses were more common than gains among primary GISTs also in the present series. The most frequent losses were of 14q (76%), 22q (44%), 1p (36%), and 15q (30%). These losses, albeit with somewhat variable rates, were reported also previously as the most common in GISTs [9, 11].

Monosomies for chromosomes 14 and 22 were cytogenetically detected early on [3, 16] and have since been accepted as highly characteristic aberrations for GISTs with both benign and malignant behavior. Yet, the pathogenetic roles of these losses, as well as of 1p and 15q, are not fully established [17–19]. Thus, it was reported [9, 11, 20, 21] that gastric and intestinal GISTs show site-dependent differences in the frequencies of the said losses. According to the proposed model, GISTs evolving along the -14q pathway are mainly gastric tumors with non-complex karyotypes and a more favorable clinical course, whereas losses of 1p and 15q are typical of intestinal tumors with more complex karyotypes and a tendency towards malignancy [11].

The present study also showed site-specific cytogenetic differences between gastric and non-gastric GISTs in the incidence of losses of 14q, 22q, 1p, 15q, and 13q. Our results are very similar to the corresponding frequencies found by CGH [11]. The higher rate we saw of the less common loss of 2p in non-gastric tumors, compared with gastric, was not reported earlier [11].

It appears that the main statistically significant differences in the cytogenetic profile between gastric and non-gastric GISTs become evident early during karyotypic evolution, when the karyotypes are still simple. In the present series, all GISTs with -14 as the sole aberration (*n* = 29) and tumors in which the -14 was the first event in clonal evolution (*n* = 10), as well as with double losses (-14, -22) (*n* = 12) and tumors in which these two changes were the first in clonal evolution (*n* = 6), were gastric. On the other hand, the high prevalence of 1p and 15q losses in non-gastric GISTs signifies their importance in site-specific, but non-gastric, clonal evolution. The evolutionary scenario in non-gastric GISTs is complicated by the fact that these tumors have no single loss, equivalent to -14, that is seen regularly and as the sole change. Nevertheless, one can hypothesize that loss of 1p plays a primary role in the tumorigenesis of non-gastric GIST, based on the data that this aberration is the most frequent and early event in karyotypic evolution. Furthermore, cases 126 and 238 of non-gastric tumors showed a single loss of 1p; sole 1p loss was also found in tumors of this location by CGH [11]. As to the next step, clones with the solitary double loss, 1p- and -15, were occasionally detected in non-gastric GISTs, not only in the present dataset (case 221) but also in reported cases [3, 11, 17]. Losses of 14q and 22q are seen in combination with 1p- and -15q at relatively high rates and also at early stages of cytogenetic evolution indicating that these four losses are nonrandomly involved in the development of non-gastric GISTs [9, 11, 20, 21].

Several CGH/array CGH studies [9, 10, 12] focused on the possible impact of mutation status - *KIT* vs. *PDGFRA* mutations - on the cytogenetic profile and progression pathways of GISTs. Among the gastric GISTs of the present series analyzed in detail, 65% carried *KIT* mutations whereas 19% had *PDGFRA* mutations. The *PDGFRA*-mutated GISTs displayed markedly fewer chromosome changes, in accordance with their generally more favorable outcome [22], even though three of them were metastatic. The frequencies of 14q and 15q losses in our study did not differ statistically between *KIT*- and *PDGFRA*-mutated gastric tumors although the rate of 22q loss in the latter was significantly lower while the rate of 1p losses was higher.

The reported data on the impact of the said mutations on the chromosome imbalance profile of the gastric GISTs are inconsistent. According to Silva et al. [10], tumors with *PDGFRA* mutations had the same overall pattern of alterations as those with *KIT* mutations but displayed less genomic complexity. Wozniak et al. [9] found no statistical difference in the frequency of any most common chromosome loss studied, such as -14q, -22q, -1p, and -15q, between *KIT*- and *PDGFRA*-mutant gastric tumors. In a systematic study of *PDGFRA*-mutated GISTs, Schaefer et al. [12] found significantly fewer chromosome aberrations in them than in *KIT*-mutated tumors. A comparison of GISTs of only gastric sites showed that losses of 14q and 22q occurred statistically less frequently in *PDGFRA*-mutated tumors.

In addition to the relative paucity of aberrations in GISTs with *PDGFRA* mutations seen in the present study, the finding of a lower incidence of 22q losses in gastric tumors with this genotype was also in accordance with published data [12]. Notably, the frequency of 22q losses (18%) in *PDGFRA-*mutated gastric GISTs found by us agrees well with the rate (15%) reported by Schaefer et al. [12]. It is of interest that in our series, none of the gastric tumors with either the double loss -14 and -22 as the only change, or this change in the basic clone, carried *PDGFRA* mutations. The importance of 22q losses in the progression of *PDGFRA-*mutated gastric GISTs is further corroborated by the fact that, in our series of totally six primary *PDGFRA-*mutated tumors with -22q, three (cases 215, 232, and 279) were high-risk and the other three (cases 37, 78a,b, and 190) were metastatic. Two of the three metastatic GISTs had nonetheless simple karyotypes and all three tumors shared losses of/in 1p, 19q, and 22q, whereas the three high-risk tumors with complex karyotypes had 1p- and -22 in common. The importance of these observations has to be evaluated on larger numbers of *PDGFRA-*mutated tumors.

Among the 33 karyotypically abnormal GISTs with *PDGFRA* mutation, two tumors had rare exon 14 and another two rare exon 12 mutations. All four had simple karyotypes. Chromosome changes in GISTs with exon 14 mutations were the same as in tumors with exon 18 mutations and typical for gastric GISTs, such as -14. Aberrations were less common in tumors with exon 12 mutations.

Among the karyotypically abnormal GISTs of the present series, *KIT* exon 9 mutations (*n* = 10) were seen mostly in non-gastric tumors, the majority of which had complex karyotypes or were metastatic. Also in another series [10], the highest cytogenetic complexity was seen in tumors bearing *KIT* exon 9 mutations. In contrast, all *KIT* exon 17 mutations (*n* = 5) were found in abnormal gastric GISTs. They displayed simple karyotypes, mainly with changes typical for this location (such as sole -14).

To our knowledge, no karyotypes of *KIT/PDGFRA* mutation-negative GISTs have been reported and existing CGH/array CGH array data on chromosome alterations in tumors with this genotype are partly inconsistent [10, 13, 23, 24]. There were 14 *KIT/PDGFRA* mutation-negative tumors in our dataset, three of which were karyotypically normal. The remaining 11 (6 gastric and 5 non-gastric) tumors comprised 5% of the 226 primary karyotypically abnormal GISTs. Three gastric GISTs (cases 39, 88, and 179) showed aberrations mainly consistent with this tumor site (such as a solitary -14), whereas the remainder displayed unusual patterns: a sole i(4)(q10) (case 228), a solitary +6 (case 226), or several polysomies (case 282). All 5 non-gastric tumors (cases 93, 94, 126, 208, and 281) had chromosome changes typical for this location. Hence, our series demonstrates a high proportion of karyotypically abnormal tumors with wild-type genotype. Some insights into the mechanisms of molecular heterogeneity of mutation-negative GISTs were provided recently [19, 25, 26].

The present study included 3 esophageal tumors (cases 57, 79, and 288). GISTs in this location are rare (1%) and, to the best of our knowledge, no karyotypic information about such tumors has been reported. The esophageal tumor of case 79 displayed changes observed in other non-gastric GISTs, such as partial deletions of 1p, 2p, 13q, and 22q. The two other tumors exhibited several trisomies, of which +4, +5, and +8 were common. The tumor of case 57 also showed concomitant losses of 1p and 2p in subclones. Surprisingly, both esophageal GISTs with trisomies (cases 57 and 288) showed the same *KIT* exon 13 mutation, Lys642Glu, whereas the tumor of case 79 had a *KIT* exon 11 mutation. The numbers are too small to say whether the karyotypic and molecular similarities between the two esophageal GISTs are coincidental or reflect something systematic.

In conclusion, the intratumor heterogeneity, including site-specific cytogenetic evolution patterns, identified in the tumors of the present series are consistent with the current concept of GISTs as a heterogeneous collection of molecular entities linked by a common histology and presumed cell of origin [27]. Based on the examination of such a large dataset, we were able to elucidate the roles of individual chromosomes in the pathogenesis of tumors with different genotypes and sites of origin, and provide further evidence that gastric and non-gastric GISTs develop via different cytogenetic pathways. Finally, karyotypically complex tumors had a more malignant behavior meaning that cytogenetic profiling could be explored as a prognostic marker, especially when applying statistical methods. A better understanding of the variable cytogenetic pathways during tumor progression may help to improve diagnostic, prognostic, and treatment decisions.

## MATERIALS AND METHODS

### Ethics statement

The study was conducted according to the guidelines of the Declaration of Helsinki, and approved by the Institutional Data Protection Officer at Oslo University Hospital (approval nr 2016/16853 and 21/03190, dated Nov. 2016 and Feb. 2021, respectively). Informed consent was obtained from all subjects involved in the study. All information about patients has been de-identified.

### Patients and tumor material

The present study comprises an unselected consecutive series of GIST samples from 291 patients received during 1998–2020. All patients were treated at Oslo University Hospital, Norway. There were 159 men and 132 women. Median age at diagnosis was 66 years (range 23–93 years). Samples from primary tumors were received from 281 patients whereas only metastases were available from 10 patients. Several samples (2 to 4) were received from 10 patients, raising the total number of samples studied to 306. 222 GISTs were located in the stomach, 48 in the small intestine, 14 in the rectum, 3 in the esophagus, and 4 were extragastrointestinal tumors. Nineteen patients (7%) had been treated with imatinib and one patient had received both imatinib and sunitinib before sampling of primary tumors; 10 patients had received imatinib before sampling of metastases (Supplementary Table 1). No treatment impact on the cytogenetic pattern was noticed.

For main analyses and correlations studies, only primary GISTs were selected. Tumors whose karyotype showed a solitary -Y (Supplementary Table 1, cases 32, 68, 86, and 272) were not included. From tumor cases 78 and 116, each represented by 2 samples, all aberrations were recorded only once.

### Chromosome banding

Fresh tissue from a representative area of the resected tumor was received and analysed cytogenetically as previously described [28].

### Mutation analyses

Genomic DNA was extracted from fresh frozen or paraffin-embedded tumor tissue. Exons 9, 11, 13, and 17 of *KIT* and exons 12, 14, and 18 of *PDGFRA* were analyzed by Sanger sequencing and categorized as described previously [29]. Tumors not analyzed in clinical routine were analyzed using AmpliSeq for Illumina Cancer Hotspot Panel version 2 as previously described [30]. The scoring was based on the sequence NM_000222.2 for *KIT* and NM_006206.4 for *PDGFRA* on the Human GRCh37/hg19 assembly [31].

### Statistical analyses

Associations between variables were investigated with two-tailed Fisher’s exact test or Pearson’s χ^2^ test for categorical variables and independent Mann-Whitney *U* test or Kruskal-Wallis test for continuous variables. *P* < 0.05 was considered statistically significant. IBM SPSS Statistics for Windows version 25.0 (Armonk, NY, USA) was used.

## SUPPLEMENTARY MATERIALS




