# Supplementary material for: Cytogenetic and molecular analyses of 291 gastrointestinal stromal tumors: site-specific cytogenetic evolution as evidence of pathogenetic heterogeneity
Source: Oncotarget. 2022 Mar 7;13:508–17. doi: 10.18632/oncotarget.28209 (PMC8901076; doi:10.18632/oncotarget.28209)
Supplement: Supplementary file 1 [file oncotarget-13-28209-s001.pdf]

Cytogenetic and molecular analyses of 291 gastrointestinal stromal tumors: site-specific cytogenetic evolution as evidence of pathogenetic heterogeneity

SUPPLEMENTARY MATERIALS

Supplementary Table 1: Cytogenetic, mutation, and clinicopathological data on 306 samples from 291 patients with GISTs. See Supplementary Table 1

Supplementary Table 2: Chromosome imbalances found in 216 primary gastric and non-gastric GISTs

	Gastric and non-gastric (*n* = 216)			
Chromosome arm	Loss[a]		Gain[a]	
	n	%	*n*	%
1p	78	36	0	0
1q	24	11	6	3
2p	19	9	11	5
2q	14	6	18	8
3p	12	6	6	3
3q	20	9	4	2
4p	18	8	12	6
4q	14	6	14	6
5p	14	6	33	15
5q	4	2	36	17
6p	10	5	10	5
6q	15	7	9	4
7p	6	3	14	6
7q	9	4	14	6
8p	13	6	23	13
8q	4	2	40	19
9p	43	20	5	2
9q	28	13	7	3
10p	23	11	3	1
10q	23	11	3	1
11p	29	13	3	1
11q	13	6	4	2
12p	17	8	11	5
12q	6	3	12	6
13q	32	15	7	3
14q	164	76	2	1
15q	64	30	2	1

16p	9	4	8	4
16q	10	5	8	4
17p	16	7	11	5
17q	14	6	13	6
18p	25	12	11	5
18q	22	10	10	5
19p	18	8	8	4
19q	25	12	11	5
20p	8	4	17	8
20q	6	3	19	9
21q	22	10	11	5
22q	94	44	1	0
Xp	18	8	2	1
Xq	19	9	2	1
Yq	12	6	5	2

[a]Partial or complete loss or gain.

Supplementary Table 3: Associations between primary tumor location and other clinicopathological variables in 226 primary GISTs

Clinicopathological variables	Tumour location		*P* value
	Gastric	Non-gastric	
Age[a]	68 (23–93)	59 (26–83)	<0.001
Sex			0.012
Female	92	13	
Male	89	32	
Tumor size (cm)[a]	5.0 (1.5–24.0)	5.8 (1.5–28.0)	0.19
Mitoses per 50 HPF[a,b]	2 (0–130)	3 (1–178)	0.15
Tumor rupture			0.034
Yes	14	8	
No	165	31	
Not determined	2	6	
Modified NIH risk criteria[c]			<0.001
Very low	6	1	
Low	80	13	
Intermediate	44	0	
High	36	22	
Metastatic	11	8	
Not able to classify	4	1	
Mutational analysis			<0.001[d]
KIT exon 9	1	7	
KIT exon 11	110	23	
KIT exon 13	1	3	
KIT exon 17	5	0	
PDGFRA exon 12	3	0	
PDGFRA exon 14	2	0	
PDGFRA exon 18	31	0	
No mutation detected	6	5	
Not done	22	7	

[a]Values are median (range). [b]HPF: high-power field of the microscope. [c]Risk classification was performed at the time of primary tumor surgery or diagnosis; NIH: National Institutes of Health. [d]*P* value is calculated based on three categories of mutations: *KIT, PDGFRA*, and no mutation.

Supplementary Table 4: Comparison of the chromosome imbalances found in primary 173 gastric and 43 non-gastric GISTs

Gastric (*n* = 173)					Non-gastric (*n* = 43)				
**Chromosome arm**	**Loss[a]**		**Gain[a]**		**Chromosome arm**	**Loss[a]**		**Gain[a]**	
	n	**%**	***n***	**%**		***n***	**%**	***n***	**%**
1p	40	23	0	0	1p	38	88	0	0
1q	17	10	3	2	1q	7	16	3	7
2p	6	3	10	6	2p	13	30	1	2
2q	8	5	16	9	2q	6	14	2	5
3p	11	6	3	2	3p	1	2	3	7
3q	17	10	2	1	3q	3	7	2	5
4p	14	8	9	5	4p	4	9	3	7
4q	11	6	10	6	4q	3	7	4	9
5p	10	6	26	15	5p	4	9	7	16
5q	4	2	26	15	5q	0	0	10	23
6p	7	4	9	5	6p	3	7	1	2
6q	10	6	8	5	6q	5	12	1	2
7p	4	2	7	4	7p	2	5	7	16
7q	8	5	7	4	7q	1	2	7	16
8p	8	5	23	13	8p	5	12	10	23
8q	2	1	31	18	8q	2	5	9	21
9p	32	18	2	1	9p	11	26	3	7
9q	21	12	4	2	9q	7	16	3	7
10p	15	9	2	1	10p	8	19	1	2
10q	17	10	2	1	10q	6	14	1	2
11p	20	12	3	2	11p	9	21	0	0
11q	6	3	3	2	11q	7	16	1	2
12p	10	6	8	5	12p	7	16	3	7
12q	4	2	8	5	12q	2	5	4	9
13q	19	11	6	3	13q	13	30	1	2
14q	137	79	1	1	14q	27	63	1	2
15q	31	18	2	1	15q	33	77	0	0
16p	7	4	6	3	16p	2	5	2	5
16q	7	4	6	3	16q	3	7	2	5
17p	12	7	9	5	17p	4	9	2	5
17q	8	5	10	6	17q	6	14	3	7
18p	14	8	8	5	18p	11	26	3	7
18q	11	6	8	5	18q	11	26	2	5
19p	13	8	6	3	19p	5	12	2	5
19q	21	12	7	4	19q	4	9	4	9
20p	4	2	12	7	20p	4	9	5	12
20q	4	2	12	7	20q	2	5	7	16
21q	14	8	7	4	21q	8	19	4	9
22q	65	38	1	1	22q	29	67	0	0
Xp	17	10	2	1	Xp	1	2	0	0
Xq	17	10	2	1	Xq	2	5	0	0
Yq	8	5	4	2	Yq	4	9	1	2

[a]Partial or complete loss or gain.

Supplementary Table 5: Comparison of the chromosome imbalances found in primary 113 gastric *KIT*-mutated and 33 gastric *PDGFRA*-mutated GISTs

Gastric *KIT*-mutated (*n* = 113)					Gastric *PDGFRA*-mutated (*n* = 33)				
Chromosome arm	Loss[a]		Gain[a]		Chromosome arm	Loss[a]		Gain[a]	
	n	%	*n*	%		*n*	%	*n*	%
1p	25	22	0	0	1p	14	42	0	0
1q	13	12	3	3	1q	2	6	0	0
2p	6	5	7	6	2p	0	0	2	6
2q	7	6	9	8	2q	1	3	6	18
3p	11	10	1	1	3p	0	0	1	3
3q	13	11	1	1	3q	2	6	0	0
4p	11	10	8	7	4p	1	3	0	0
4q	11	10	8	7	4q	0	0	0	0
5p	7	6	21	19	5p	3	9	0	0
5q	4	4	21	19	5q	0	0	0	0
6p	6	5	6	5	6p	0	0	1	3
6q	9	8	5	4	6q	0	0	1	3
7p	4	4	7	6	7p	0	0	0	0
7q	5	4	7	6	7q	0	0	0	0
8p	4	4	16	14	8p	4	5	2	6
8q	1	1	20	18	8q	1	3	5	15
9p	24	21	1	1	9p	3	9	0	0
9q	20	18	2	2	9q	0	0	1	3
10p	13	12	1	1	10p	0	0	0	0
10q	15	13	1	1	10q	1	3	0	0
11p	12	11	2	2	11p	3	9	0	0
11q	5	4	2	2	11q	0	0	0	0
12p	5	4	6	5	12p	3	9	0	0
12q	3	3	5	4	12q	1	3	0	0
13q	11	10	5	4	13q	4	12	0	0
14q	95	84	0	0	14q	24	73	1	3
15q	25	22	2	2	15q	3	9	0	0
16p	5	4	4	4	16p	0	0	1	3
16q	6	5	4	4	16q	1	3	1	3
17p	4	4	8	7	17p	4	12	0	0
17q	6	5	8	7	17q	2	6	1	3
18p	6	5	7	6	18p	5	15	1	3
18q	6	5	7	6	18q	2	6	1	3
19p	10	9	5	4	19p	2	6	0	0
19q	14	12	6	5	19q	6	18	0	0
20p	1	1	10	9	20p	2	6	0	0
20q	2	2	10	9	20q	2	6	0	0
21q	10	9	6	5	21q	3	9	0	0
22q	48	43	1	1	22q	6	18	0	0
Xp	15	13	1	1	Xp	1	3	1	3
Xq	15	13	1	1	Xq	1	3	1	3
Yq	8	7	4	4	Yq	0	0	0	0

[a]Partial or complete loss or gain.
